# A Multi-Block Multivariate Analysis to Explore the Influence of the Somatic Maturation in Youth Basketball

**DOI:** 10.3389/fpsyg.2021.602576

**Published:** 2021-02-01

**Authors:** Jorge Arede, Irene Oliveira, Miguel-Angel Ángel Gomez, Nuno Leite

**Affiliations:** ^1^Research Centre in Sports Sciences, Health Sciences and Human Development, Vila Real, Portugal; ^2^Department of Mathematics, University of Trás-os-Montes and Alto Douro, Vila Real, Portugal; ^3^CEMAT-IST-UL, Center for Computational and Stochastic Mathematics, University of Lisbon, Lisbon, Portugal; ^4^Faculty of Physical Activity and Sport Sciences, Polytechnic University of Madrid, Madrid, Spain

**Keywords:** talent, development, puberty, performance analysis, identification, growth, maturation, adolescence

## Abstract

The aim of this study was to examine the influence of somatic maturation in anthropometric, physical, and game-related variables in youth basketball age groups under-13 (U-13) and under-15 (U-15). One-hundred and eighty-five basketball players performed anthropometrical and physical tests during a non-official youth basketball tournament. Predicted maturity offset (MO) and game-related variables were also analyzed. Cluster analysis was used to analyze the between-maturation status differences in all parameters in each age group. Also, regularized generalized canonical correlation analysis (RGCCA) was used to assess relative contributions of maturational, physical, and game-related variables within each age group. Based on MO, two different clusters were identified within each age category. Greater differences in MO were identified among U-13 clusters than among U-15 clusters. No significant differences were observed between clusters in terms of physical and game-related variables. High correlations between maturational, physical, and game-related variables (i.e., points scored, field goals attempted, and rebounds) were found for boys. In girls, different trends in terms of correlations were observed. The strongest association between blocks was observed between physical tests and game-related variables in all age categories, except for U-15 girls. Knowing and identifying performance profiles according to biological age is of upmost importance since it allows the coach to create challenging situations adjusted to the individual’s needs.

## Introduction

Maturation refers to the developmental process toward the adult or mature state and can be defined in terms of status, tempo, and timing (Malina et al., 2004; Malina et al., 2015, Malina, 2016). The study of this individual’s variation effect in growth and maturation has contributed to a better understanding of its influence in different areas of development, such as physical and technical parameters (Malina et al., 2004; Baxter-Jones, 2009; Coelho e Silva et al., 2010; Sherar et al., 2010). One of the best ways to improve the understanding about players’ development is based on the establishment of different maturity timing categories, according to the age of peak height velocity (APHV), in comparison to average of population (Sherar et al., 2005), but also on different maturity status based on years to APHV [i.e., maturity offset (MO)] (Carvalho et al., 2017). During adolescence, body mass and physical abilities play a decisive role in performance, and may positively influence the participation in youth competitions (Malina et al., 2004). However, evidence emerging from longitudinal studies seems to confirm that young players who experience the greatest physical challenges, as long as they are feasible, become more resilient and demonstrate a superior ability to develop the technical and psychological attributes required for adult professional success (Ostojic et al., 2014). Specifically, girls showed divergent patterns of physical development and differences in physical performance are not as significant as in boys (Baxter-Jones et al., 2002; Sherar et al., 2010). Based on this rationale, the importance of future research when considering the effect of maturation on the development of youth players (e.g., basketball) in both genders can be derived.

Usually, the sports system includes chronological age (CA)-based training and competitions, using the “one-size-fits-all” approach, instead of considering the biological age (i.e., bio-banding), which could challenge the athlete in a unique way and create a learning environment that is more diversified and suited to the physical, technical, and tactical development of each player (Cumming et al., 2017; Arede et al., 2019). During the adolescence, there are inter-individual differences at biological level within each age category, which may lead to distinct performance in other domains (Coelho e Silva et al., 2010; Arede et al., 2019). Using the pubic hair status as a method to estimate biological maturation of youth basketball players, in under-13 (U-13) level most of the players were in puberty (46%), while in under-14 (U-14) level were in late puberty (56%) (Coelho e Silva et al., 2010). Additionally, in each age category, other maturity factors were identified. In particular, Arede et al. (2019) using the predicted MO approach (Mirwald et al., 2002) found distinct maturity status within the same age category in the under-15 (U-15) boys national team. The authors clustered players into pubertal (MO = 0.31 ± 0.41, *n* = 9), late pubertal (MO = 0.97 ± 0.21, *n* = 10), and in post pubertal (MO = 1.73 ± 0.21, *n* = 15) groups. These distinct maturity statuses (i.e., clusters) underpin between-group differences at physical and game performance levels. For example, pubertal players had higher aerobic fitness than late pubertal and post pubertal players, but late pubertal players outperformed their counterparts in upper body power (Arede et al., 2019). The game performance results showed that post-pubertal players outscored in blocks, but pubertal players were better in assists, assist/turnover ratio (Ast:TO), and steal/turnover ratio (Stl:TO) (Arede et al., 2019). However, biological maturation needs to be controlled for sex effect due to the fact that it has important differences between adolescent males and females (Drinkwater et al., 2008), and more research is required for a better understanding of maturation effect on each age category. Females tend to reach their PHV at approximately 12 years old, and have shorter growth period, whereas males reach their PHV at 14 years old, with greater velocity curves of peak height and body mass (Sherar et al., 2005). However, there is little research accounting for how biological age differs in each age category in youth basketball (particularly in females), but also how different domains are related, including game-related variables.

Specifically, relationships between different maturation parameters (biological maturation, physical, and game-related variables) were found at the U-14 boys national level basketball (Torres-Unda et al., 2013). The MO was positively correlated to point average and negatively correlated to 20-m sprint test (20-m sprint), and Abalakov jump (ABA). Moreover, 20-m sprint, and ABA were significantly negatively correlated to point average. The same authors also explored the interaction between domains, and they concluded that MO and stature in conjunction with ABA, as well as stature in conjunction with 20-m sprint were significantly positively correlated (Torres-Unda et al., 2013). In fact, more extensive analysis including canonical correlation analysis had been used to examine the relationships between multivariate domains, and measured the magnitude of the association between variables in youth players (Coelho e Silva et al., 2010; Matthys et al., 2012). In particular, significant relationships were identified between APHV, height, and weight; and between handgrip, 20-m sprint, and five-jump test (Matthys et al., 2012). Thus, more mature players were taller, heavier, and presented better performance in physical tests, but not on sport-specific skills (Matthys et al., 2012). In other study, in youth basketball, functional capacities and basketball skills showed substantial positive correlations with 20-m shuttle run, and sit-ups as primary contributors to performance in sport-specific skills (Coelho e Silva et al., 2010). However, there is need of bridging the gap between science and practice helping the practitioners to increase their effectiveness of their decisions during training and competitions, and then grouping players according to growth and maturation attributes (Cumming et al., 2017), particularly in the case of knowing the relationships of maturational and physical domains with game-related variables.

Therefore, considering the need to provide some recommendations to help coaches to improve the development of young basketball players, the aim of this study was to examine the influence of maturation in anthropometric, physical, and game-related variables in youth boys’ and girls’ basketball age groups of U-13 and U-15. The hypothesis of the study suggests that physical and game-related variables are different by sex and largely associated with biological maturation in both age groups.

## Materials and Methods

### Subjects

One-hundred and eighty-five basketball players taking part in a U-13 boys’ category (mean CA = 12.91 ± 0.56, *n* = 50), U-13 girls’ category (mean CA = 12.75 ± 0.70, *n* = 29), U-15 boys’ category (mean CA = 14.77 ± 0.54, *n* = 54), and U-15 girls’ category (mean CA = 14.42 ± 0.78, *n* = 52) were considered for this study (Table 1). The U-13 age category included those players born before or within the year 2005, while U-15 age category included players born on the years 2004 and 2003. Most players were of Portuguese Ancestry, apart from few players of African (*n* = 3), Eastern Europe (*n* = 1), and South American Ancestry (*n* = 1). All participants were selected by sport clubs to play in a non-official youth basketball tournament, which included 10 boys’ teams and nine girls’ teams. During this 3-day event, every team played a minimum of six and a maximum of seven games. The eligibility criteria of the study were: (i) players who did not suffer any injury during the last 6 months (including tournament); (ii) players who took part in at least four matches during the tournament; and (iii) those players who completed the full test battery. From the original sample (*n* = 198), 14 participants were excluded because due to incomplete full test (U-13 boys, *n* = 6; U-13 girl, *n* = 2; U-15 boys, *n* = 3; U-15 girls, *n* = 2). Written informed consent was obtained from all participants and their parents before the investigation began. The present study was approved by the University of Trás-os-Montes and Alto Douro research ethics committee and conformed to the recommendations of the Declaration of Helsinki.

**TABLE 1:** Descriptive and inferential analysis of anthropometrical and maturational variables and blocks, according to the age groups and established clusters (Mean ± SD).

		BOYS				GIRLS				Magnitude-based inferences			
Blocks	Variables	U-13		U-15		U-13		U-15		U-13	U-15	U-13	U-15
		Pre-PHV C1 (*n* = 29)	Mid-PHV C2 (*n* = 21)	Mid-PHV C3 (*n* = 27)	Post-PHV C4 (*n* = 27)	Mid-PHV C1 (*n* = 7)	Post-PHV C2 (*n* = 22)	Post-PHV C3 (*n* = 17)	Post-PHV C4 (*n* = 35)	Boys C1–C2	Boys C3–C4	Girls C1–C2	Girls C3–C4
Anthropometrical	CA (y)	12.7 ± 0.6	13.2 ± 0.4	14.7 ± 0.6	14.9 ± 0.5	12.1 ± 0.6	12.9 ± 0.6	13.9 ± 0.8	14.7 ± 0.6				
	Height (cm)	154.4 ± 4.7	166.7 ± 4.7	167.6 ± 8.5	178.7 ± 5.8	153.7 ± 5.9	162.8 ± 4.9	158.3 ± 2.4	166.7 ± 5.5				
	Body mass (kg)	42.8 ± 6.2	57.9 ± 8.9	54.4 ± 5.2	68.6 ± 5.0	38.1 ± 4.2	57.8 ± 9.9	52.3 ± 4.7	62.3 ± 6.7				
	%BF (%)	12.7 ± 4.7	15.2 ± 6.0	10.3 ± 2.7	13.2 ± 3.8	16.9 ± 4.3	26.9 ± 7.2	24.5 ± 4.4	27.0 ± 5.5				

Maturational	MO (y)	−2.2 ± 1.0	−0.5 ± 0.4	0.2 ± 1.0	1.2 ± 0.5	−0.2 ± 0.5	1.2 ± 0.4	1.4 ± 0.4	2.3 ± 0.4	−2.41** [−3.01;−1.81]	−0.72** [−1.41;−0.03]	−2.10** [−21.81; 17.61]	−2.11** [−2.90;−1.31]
	APHV (y)	14.9 ± 1.1	13.7 ± 0.5	14.4 ± 0.9	13.7 ± 0.4	12.3 ± 0.3	11.7 ± 0.4	12.5 ± 0.4	12.3 ± 0.4	1.60** [1.00; 2.21]	1.06 [0.44; 1.68]	1.58** [0.78; 2.39]	0.32 [−0.28; 0.92]
	PAH (cm)	183.7 ± 4.3	187.3 ± 3.2	180.4 ± 5.9	185.9 ± 4.9	169.1 ± 4.8	169.3 ± 4.9	162.9 ± 4.2	168.6 ± 5.4	−0.99 [−1.57; −0.41]	−1.00 [−1.54; −0.46]	−0.02 [−0.90; 0.86]	−1.14** [−1.71;−0.58]

Note: **p < 0.01. Abbreviations: CA, chronological age; MO, maturity offset; APHV, age of peak height velocity; PAH, predicted adult height; %BF, body fat; C1, Cluster 1; C2, Cluster 2; C3, Cluster 3; C4, Cluster 4. Cohen’s “d” units, and threshold values were: 0–0.2 trivial; >0.2–0.6 small; >0.6–1.2 moderate; >1.2–2.0 large; and >2.0.

### Procedures

All data were gathered during a 3-day non-official youth basketball tournament, using cross-sectional design. The data collection followed a fixed schedule and supervised by the same members of the research team ensuring the test accuracy and reliability. In the morning of day 1, anthropometrical data were collected. Then, in early afternoon of the same day, physical measurements were performed in indoor basketball court with verbal encouragement. Specific warm-up was performed prior to the physical tests (Taylor et al., 2013). The protocol included a cardiovascular phase (5 min of general jogging/run), followed by dynamic stretching and task-specific activity, which consisted of two 20-m slalom runs, two 40-m shuttle sprints at 50 and 75% of the subjects’ perceived maximal effort, respectively, and one maximal 40-m sprint (Taylor et al., 2013). A practical demonstration and familiarization (minimum two trials) with testing procedures were held before the tests were conducted. Finally, game-related variables were collected during non-official youth basketball tournament games (late afternoon of day 1, day 2, and day 3).

### Anthropometrical Data

Height and seated height were recorded for estimation of MO, using portable stadiometer (Tanita BF-522W, Japan, nearest 0.1 cm), and following specific guidelines (Taylor et al., 2013). Body mass was estimated using the body fat (BF) monitor (Tanita BF-522W, Japan, nearest 0.1 kg). All measurements were taken following the guidelines outlined by the International Society for the Advancement of Kinanthropometry (ISAK). In addition, the %BF was estimated using the same device, through the bioelectrical impedance analysis technique (intraclass correlation coefficient [ICC] = 0.87, [95% CI 0.47; 0.97]; typical error of measurement [TEM] = 0.44, [95% CI 0.29; 0.89]). According to the manufacturer instructions, the present BF monitor is suitable for adults and children (ages 7–17) with inactive to moderately active lifestyles and adults with athletic body types. Height and seated height were assessed while subjects were barefoot and gathered by the same researcher to ensure testing accuracy and reliability. The ICC for Height was 0.97 (95% CI 0.85; 0.99) and for TEM was 0.23 (95% CI 0.15; 0.46). The ICC for Seated Height was 0.90 (95% CI 0.58; 0.98) and for TEM was 0.39 (95% CI 0.26; 0.79).

### Somatic Maturation

Despite having some limitations and although new prediction equations are appearing in these last years, the assessment of the years from/to the peak height velocity (PHV) (i.e., predicted MO) is the most commonly used indicator of the somatic maturation in the sports field (Kozieł and Malina, 2018). The MO was estimated using a non-invasive method appropriated for age range of sample, considering anthropometric data (leg length and sitting height) and CA, which has shown to be accurate with a reported error of approximately 6 months (Mirwald et al., 2002). This method has previously been tested in longitudinal study among 8–18 years old boys and girls (Malina and Kozieł, 2014a,b). Leg length was estimated as the difference between height and sitting height (Mirwald et al., 2002). The APHV was calculated by subtracting the MO from the CA (Mirwald et al., 2002). Considering the MO, the subjects could be classified into three maturity status categories: pre-PHV (PHV ≤ –1.00 year), mid-PHV (−1.00 < PHV < +1.00 year), and post-PHV (PHV ≥ +1.00) (Carvalho et al., 2017). Predicted adult height (PAH) resulted from the sum of the height at the time of the measurements and distance left to grow in height according to APHV and MO (Sherar et al., 2005). No published data exist on the validation of equation to predict adult height. Consequently, we used unpublished data from our laboratory using male basketball national team players (*n* = 19) recorded an underestimated adult height at 16 years old using non-invasive method (Sherar et al., 2005) comparing with real value at adult age [*p* = 0.007; mean difference (SD) between methods = −1.86 ± 0.63 cm, 95% CI = −3.19 to −0.69). However, the data recorded almost perfect relationship and substantial agreement between the values of PAH measured with both methods [*r* = 0.93, 95% CI = 0.88–0.98, standard error of measurement (SEE) = 0.03 cm, slope of the regression line = 0.86; *P* < 0.001, ICC = 0.93, 95% CI = 0.83–0.97, Cronbach’s alpha (α) = 0.96]. Also, unpublished data from our laboratory (male population) shown that the Mirwald method (*n* = 15; CA range = 12.20–15.43 years) significantly overestimated adult height comparing with gold-standard Khamis and Roche method [*p* = 0.003; mean difference (SD) between methods = 3.77 ± 0.91 cm, 95% CI = 2.14–5.70]. However, the data recorded almost perfect relationship and substantial agreement between the values of PAH measured with both methods [*r* = 0.92, 95% CI = 0.74–0.97, SEE = −0.06 cm, slope of the regression line = 0.83; *P* < 0.01, ICC = 0.91, 95% CI = 0.76–0.97, Cronbach’s alpha (α) = 0.96]. Based on clustering analysis (see section “Statistical Analysis”), participants were grouped considering MO for each age category as follows: U-13 boys more mature vs less mature players (clusters 1 [C1], and 2 [C2]), and the procedure was repeated for U-15 boys (clusters 3 [C3] and 4 [C4]), as well as to girls’ age groups, U-13 more mature vs less mature players (clusters 1 [C1] and 2 [C2]), and U-15 (clusters 3 [C3] and 4 [C4]). Considering the difference to the mean APHV in reference population subjects could be classified into three maturity timing categories: early (APHV ≤ −1.00 years), average (−1.00 < APHV < +1.00 years), and late (APHV ≥ +1.00 years) (Sherar et al., 2005). Most players were average maturing, apart from few players who were early maturing (C3 boys = 1; C4 boys = 2; C2 girls = 1) or late maturing (C1 boys = 8; C2 boys = 4; C3 boys = 5; C3 girls = 1; C4 girls = 1).

### Testing

Jump ability was recorded using an infrared optical system (*OptoJump Next—Microgate, Bolzano, Italy*). Running and change-of-direction speed were recorded with 90-cm height photoelectric cells separated by 1.5 m (Witty; Microgate). Each participant performed two trials of jumping and running abilities with 2 min of rest between the trials. Players started each speed tests in standing position with their foot 0.5 m behind the first timing gate.

### Jumping Ability

Vertical jumps [ABA and horizontal jump (HJ)] were performed according to the protocol described by Bosco et al. (1983). The ICC for ABA was 0.97 (95% CI 0.84; 0.99) and for TEM was 0.23 (95% CI 0.15; 0.51). The ICC for HJ was 0.89 (95% CI 0.49; 0.98) and for TEM was 0.42 (95% CI 0.27; 0.92).

### Speed and Agility

Speed was evaluated by 20-m sprint (ICC = 0.96, [95% CI 0.83; 0.99]; TEM = 0.24, [95% CI 0.16; 0.49]) and the agility *t*-test determined speed with directional changes (forward sprinting, lateral shuffling, and backward running) (ICC = 0.82, [95% CI 0.33; 0.96]; TEM = 0.50, [95% CI 0.33; 1.01]) (Pauole et al., 2000).

### Game-Related Statistics

Game-related variables (points, field-goals attempted, total rebounds, assists, steals, and turnovers) during the youth tournament were used to assess players’ performances (see Table 1). All data were collected using the official FIBA box-scores recorded by experienced and qualified technicians. All calculations were performed as per National Basketball Association procedures. All game-related variables were presented as a percentage of the team total score (Arede et al., 2019). The Ast:TO and Stl:TO ratios were calculated by comparing the number of assists or steals to the number of turnovers committed (Arede et al., 2019).

### Statistical Analyses

Data were presented as mean ± standard deviation. First, to analyze data normality assumptions, the Shapiro–Wilk test was used. Then, in cases of non-Gaussian distribution, non-parametric methods were used to evaluate differences between medians. Second, a two-step cluster analysis was performed using Ward’s method—squared Euclidian distance as a distance measure—considering MO as the grouping variable (Everitt, 2011). Third, one-way ANOVA with Bonferroni *post hoc* analyses were conducted to make comparisons between clusters. Effect sizes (ES) of the difference were assessed using standardized Cohen’s “*d*” units, and threshold values were: 0–0.2 trivial; >0.2–0.6 small; >0.6–1.2 moderate; >1.2–2.0 large; and >2.0 very large (Batterham and Hopkins, 2006). Lastly, to assess the relative contributions of maturational, physical, and game-related variables, the regularized generalized canonical correlation analysis (RGCCA) was used with scaled variables. In this study, RGCCA was used to extract the information shared by the blocks of variables, namely, maturational, physical, and game-related which were all linked through the reduction to a few meaningful canonical variables (CV), visualization of relevant variables, and interaction between these blocks. It was decided to extract and evaluate a pair of CV (CV_1_ and CV_2_), since we aim to extract the maximum information of the interrelations within and between the three blocks of variables. The statistical package IBM SPSS 24.0 (Armonk, NY, United States: IBM. Corp.) was used to perform the statistical tests and cluster analysis. RGCCA analysis was conducted using “RGCCA” package in R 3.4.2 (R Core Team, 2014) (Tenenhaus and Guillemot, 2017).

## Results

Tables 1, 2 display the descriptive analysis of anthropometrical, maturational, physical, and game-related variables for each age group according to the established clusters.

**TABLE 2:** Descriptive and inferential analysis of physical and game-related variables and blocks, according to the age groups and established clusters (Mean ± SD).

Blocks	Variables	BOYS				GIRLS				Magnitude-based inferences			
		U-13		U-15		U-13		U-15		U-13	U-15	U-13	U-15
		Pre-PHV C1 (*n* = 29)	Mid-PHV C2 (*n* = 21)	Mid-PHV C3 (*n* = 27)	Post-PHV C4 (*n* = 27)	Mid-PHV C1 (*n* = 7)	Post-PHV C2 (*n* = 22)	Post-PHV C3 (*n* = 17)	Post-PHV C4 (*n* = 35)	Boys C1–C2	Boys C3–C4	Girls C1–C2	Girls C3–C4
Physical	20m (s)	3.7 ± 0.2	3.6 ± 0.3	3.3 ± 0.2	3.2 ± 0.2	3.8 ± 0.1	3.8 ± 0.6	3.6 ± 0.1	3.7 ± 0.1	0.27 [−0.34; 0.88]	0.51 [−0.02; 1.05]	−0.02 [−1.00; 0.95]	−0.32 [−0.88; 0.25]
	T-test (s)	11.3 ± 0.6	11.4 ± 0.9	10.3 ± 0.6	9.9 ± 0.6	12.4 ± 0.5	11.8 ± 0.7	11.3 ± 0.5	11.6 ± 0.6	−0.16 [−0.78; 0.45]	0.50 [−0.03; 1.30]	0.89 [0.07; 1.70]	−0.48 [−1.05; 0.08]
	ABA (cm)	29.4 ± 6.2	30.0 ± 5.9	38.4 ± 5.8	39.9 ± 5.3	26.5 ± 2.4	26.2 ± 4.0	27.8 ± 3.1	26.5 ± 4.5	−0.11 [−0.67; 0.44]	−0.27 [−0.81; 0.27]	0.17 [−0.63; 0.96]	0.41 [−0.16; 0.98]
	HJ (cm)	162.8 ± 21.6	162.1 ± 21.4	198.7 ± 21.7	211.2 ± 23.1	150.7 ± 12.1	148.6 ± 18.2	152.7 ± 10.0	154.6 ± 16.2	0.02 [−0.53; 0.58]	−0.52 [−1.05; 0.01]	0.17 [−0.63; 0.96]	−0.10 [−0.68; 0.47]
Game-related statistics	Points (n)	7.8 ± 7.2	12.2 ± 11.8	6.8 ± 7.8	11.2 ± 11.0	9.6 ± 11.7	14.4 ± 12.9	9.8 ± 10.4	8.7 ± 7.4	0.01 [−0.68; 0.69]	−0.39 [−0.97; 0.19]	−0.27 [−1.36; 0.82]	−0.05 [−0.69; 0.80]
	Field goals (n)	3.3 ± 2.0	4.0 ± 3.1	3.0 ± 2.2	3.8 ± 2.8	3.4 ± 2.2	3.4 ± 2.2	3.6 ± 1.9	3.1 ± 1.8	−0.06 [−0.64; 0.52]	−0.22 [−0.76; 0.32]	−0.68 [−1.92; 0.56]	0.44 [−0.14; 1.02]
	Rebounds (n)	7.2 ± 6.3	9.9 ± 8.3	6.7 ± 5.6	9.9 ± 6.1	9.1 ± 4.7	13.4 ± 9.4	8.1 ± 6.5	9.9 ± 6.7	−0.51 [−1.10; 0.08]	−0.73 [−1.30; −0.16]	−0.30 [−1.15; 0.55]	−0.23 [−0.84; 0.38]
	Ast:TO (a.u.)	0.2 ± 0.3	0.1 ± 0.2	0.2 ± 0.3	0.4 ± 1.1	0.1 ± 0.1	0.2 ± 0.4	0.2 ± 0.3	0.2 ± 0.5	−1.32 [0.39; 2.26]	0.12 [−0.81; 1.04]	−0.85 [−1.96; 0.25]	−0.10 [−0.97; 0.77]
	Stl:TO (a.u.)	0.8 ± 1.5	0.5 ± 0.7	0.5 ± 0.5	1.0 ± 1.5	0.3 ± 0.3	0.9 ± 0.8	0.9 ± 1.0	0.9 ± 1.3	0.13 [−0.56; 0.82]	−0.62 [−1.22; −0.02]	−0.81 [−1.80; 0.18]	0.03 [−0.60; 0.66]

*Abbreviations: 20m, 20 meters’ sprint time; T-test, agility *t*-test; ABA, Abalakov vertical jump; HJ, horizontal jump; Ast:TO, assist per turnover ratio; Stl:TO, steal per turnover ratio; C1, Cluster 1; C2, Cluster 2; C3, Cluster 3; C4, Cluster 4. Cohen’s “d” units, and threshold values were: 0–0.2 trivial; >0.2–0.6 small; >0.6–1.2 moderate; >1.2–2.0 large; and >2.0.*

**FIGURE 1:**
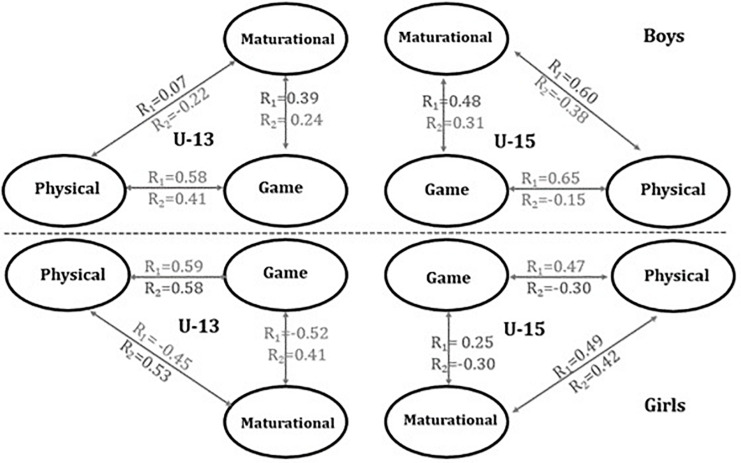
Canonical variables correlations between maturational, physical, and game-related statistics blocks (R_*cv1*_ and R_*cv2*_). Abbreviations: U-13, under-13; U-15, under-15.

In both gender, the results show significant differences in the APHV and MO between C1 and C2 (U-13) and between C3 and C4 (U-15) (all *p* < 0.01). In both genders, subjects included in C1 and C4 presented a later APHV, comparing to their peers of same age category. The Post-PHV U-15 boys (C4) had higher values in speed, agility, and lower limb power tests. Within each age category, more mature subjects generally scored more points, attempted more field goals, and got more rebounds, but not significantly. The results of the correlation matrix among canonical variates are displayed in Figure 1. The U-13 boys showed the highest correlation between physical and game-related variables followed by maturational and game variables. The U-15 boys showed the highest correlation between physical and game-related variables, followed by physical and maturational variables. The U-13 girls showed the highest correlation between physical and game-related variables, followed maturational and game-related variables; in CV_2_, the highest correlation was found between game-related statistics and physical. The U-15 females showed the highest correlation between maturational and physical variables, followed by physical and game-related variables.

**FIGURE 2:**
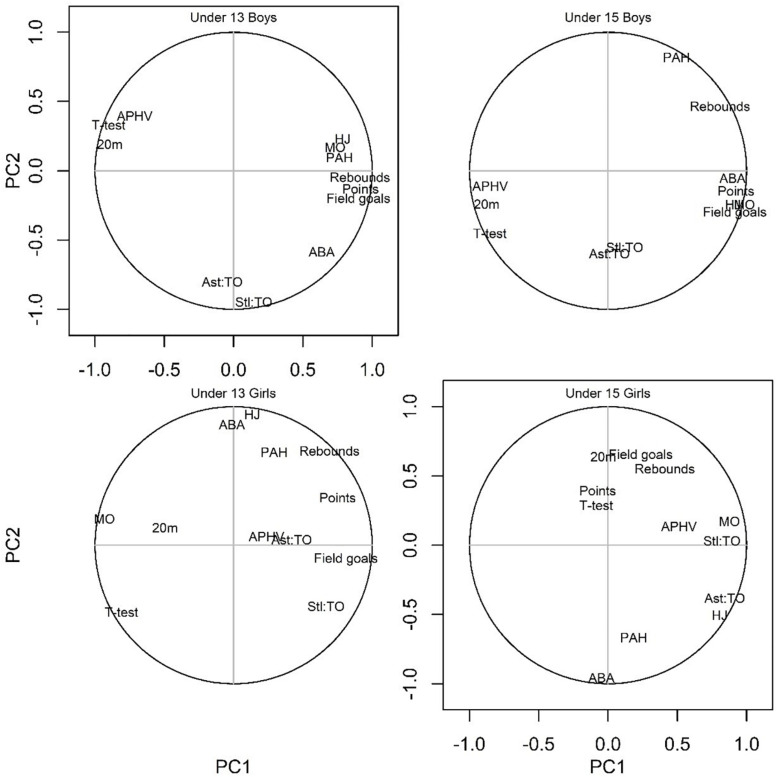
Graphical representation for correlations between original and canonical variables within groups. Legend: MO, maturity offset; APHV, age of peak height velocity; PAH, predicted adult height; %BF, body fat; 20m, 20 meters’ sprint time; T-test, agility *t*-test; ABA, Abalakov vertical jump; HJ, horizontal jump; Ast:TO, assist per turnover ratio; Stl:TO, steal per turnover ratio; PC1, principal component 1; PC2, principal component 2.

Table 3 and Figure 2 show correlations between original and CVs within age groups for boys and girls. In U-13 and U-15 boys, the results confirmed that players with higher MO, higher PAH, and lower APHV also had high scores in physical variables, and scored more points, field goals attempted, and rebounds. Moreover, the results of CV_2_ in U-13 boys revealed that players with higher APHV presented lower performance in Ast:TO and Stl:TO ratios. Considering the U-13 girls, the results of CV_1_ demonstrated that players with lower MO had low performance in sprinting and agility test, but also in all game-related statistics. The results of CV_1_ for the U-15 girls showed that players with higher MO and APHV had better HJ, got more rebounds, and presented better game efficiency performance (i.e., Ast:TO and Stl:TO ratios). Moreover, the results of CV_2_, demonstrated that players with higher PAH had better in ABA and HJ, attempted lower field goals, scored less points, got less rebounds, but had better Ast:TO ratio.

**TABLE 3:** Correlations between original and canonical variables within groups for boys’ and girls’ age groups.

Original variables	BOYS				GIRLS			
	U-13		U-15		U-13		U-15	
	CV_1_	CV_2_	CV_1_	CV_2_	CV_1_	CV_2_	CV_1_	CV_2_
MO	0.753	0.156	0.970	−0.243	−0.957	0.191	0.877	0.173
APHV	−0.713	0.396	−0.850	−0.109	0.238	0.064	0.512	0.137
PAH	0.763	0.125	0.494	0.821	0.292	0.674	0.185	−0.665
20m	−0.893	0.192	−0.876	−0.240	−0.495	0.128	−0.034	0.640
T-test	−0.902	0.331	−0.853	−0.450	−0.810	−0.482	−0.085	0.289
ABA	0.637	−0.585	0.899	−0.053	−0.012	0.871	−0.046	−0.956
HJ	0.786	0.231	0.942	−0.259	0.135	0.944	0.806	−0.506
Points	0.916	−0.130	0.924	−0.185	0.751	0.344	−0.074	0.394
Field goals	0.901	−0.206	0.915	−0.305	0.810	−0.102	0.237	0.647
Rebounds	0.911	−0.046	0.808	0.466	0.692	0.682	0.409	0.552
Ast:TO ratio	−0.082	−0.801	0.010	−0.578	0.419	0.041	0.843	−0.381
Stl:TO ratio	0.146	−0.946	0.120	−0.551	0.667	−0.440	0.822	0.032

*MO, maturity offset; APHV, age of peak height velocity; PAH, predicted adult height; Abbreviations: 20m, 20 meters’ sprint time; T-test, agility *t*-test; ABA, Abalakov vertical jump; HJ, horizontal jump; Ast:TO, assist per turnover ratio; Stl:TO, steal per turnover ratio; CV1, canonical variable 1; CV2, canonical variable 2.*

## Discussion

The main objective of this cross-sectional study was to examine the influence of the MO on the composition of U-13 and U-15 basketball age groups in both genders. As was argued, the age group, gender, and maturational factors may affect the performances displayed by players. The current findings support this hypothesis due to the fact that the MO allowed establishing two different cohorts within each age group. Globally, the results confirm the influence of MO in performance profiling of youth basketball players. Moreover, the correlation between maturational, physical, and game-related variables correlated differently according to age category, and gender. Evidence regarding the differences that usually characterize players of the same age group in terms of anthropometrical scores, body composition, and physical measurements from previous cross-sectional research in youth team ball sports was confirmed in this study (Malina et al., 2004; Malina et al., 2007). More mature players are typically characterized by higher performance in speed, agility, and lower limb power which are determinants of basketball and positively influence the probability of being selected to practice and play with the best players and coaches (Sherar et al., 2010). Although the results of this study indicate that more mature players possessed no significant physical or game advantages, the MO variation between clusters clearly indicates the existence of different maturational statuses within different age categories. Evidence from a recent study with young basketball players (Arede et al., 2019) are in accordance with our results, especially the non-significant differences between maturity status in points scored and game efficiency. However, it is important to note that data from the present study were obtained from a sample consisting of participants aged between 11 and 15 years old, contrary to what occurred in the previous studies (Lefevre et al., 1990; Malina et al., 2007; Arede et al., 2019), where the samples were less numerous and shorter regarding to the age ranges. Particularly important in the case of girls’ subsets was that the results showed a more homogenous anthropometric and physical profile than in boys’ sample. Maturity-related trends in anthropometric and physical performance of girls are consistent with those for boys, but differences in physical capacities are less apparent. Limited data suggest that less mature girls perform better than more maturing girls in some tasks, but overall maturity-associated variation is not consistent across tasks and ages (Myburgh et al., 2016).

A greater difference was identified when comparing game-related variables between U-13 clusters (C2–C1) than between U-15 clusters (C4–C3). Additionally, the differences between maturation-related clusters are different according to gender. In fact, between-gender differences in players’ development in youth basketball were previously reported with girls revealing lesser commitment to sport (lower average minutes per week and per season) (Leite and Sampaio, 2012). Thus, the development of motor abilities, as well as technical–tactical performances may deteriorate, contributing to different between maturity status group discrepancies than in boys. In both age categories, boys in advanced maturity status scored more points and got more rebounds. Although non-significant, the comparison of game-related variables revealed interesting trends: on the one hand, the analysis of the variation between clusters of the same age group (i.e., C1–C2 and C3–C4) and on the other hand, the between-gender variation. Globally, more mature players were characterized by a higher contribution to team’s performance in terms of points scored, rebounds, and field-goals attempted. These game actions are highly dependent on conditional motor skills, such as strength, speed, or power, which aid more mature players in obtaining better scores (Torres-Unda et al., 2013). However, they also depend on the game knowledge, i.e., perceptual-cognitive skills. Along these lines, the results are contradictory to previous findings (Arede et al., 2019), since ratio scores were not significantly different across maturity status. Improved ball-handling and passing (i.e., assists) and gain possession (i.e., steals) skills are strongly associated with the speed in which multiple objects may be visually tracked, namely, visual tracking speed (Mangine et al., 2014). Thus, the visual-motor capability, which elicits better game-related performance, may be associated with better pattern recognition skills due to a higher number of hours dedicated to sport-specific practice and competition (Arede et al., 2019). Besides, assists and steals are strongly associated with agility skills (McGill et al., 2012). In the present study, the more mature players obtained better records in agility than those who were less mature. Higher body control in different planes may not only contribute to increasing the level of defensive pressure but also promote high-quality distribution skills. Thus, due to the biological advantage, the more mature may benefit from both training experience in affordable situations to correctly respond to the recognized game patterns and easily transfer to game situations.

The results of the present study confirm the existence of different maturity status among U-13 and U-15 boys and girls. From a methodological point of view, the maturity status assessment can be supported in isolated or combined criteria (Lloyd et al., 2014), but it is more important to create the proper competitive environment to foster talent in sport. Coaches recognize the importance of grouping athletes relative to attributes associated with the processes of maturation in youth sport and because they are forced to reflect on other players, provides them the opportunity to evaluate skills and attributes in a more balanced environment (Sherar et al., 2005; Cumming et al., 2017; Malina et al., 2019). Considering the variation in the MO that characterized participants in the youth basketball tournament, the results should be cautiously used when applying a competitive model, since it is clear that players had distinct body composition and physical scores may be competing for the same sport in a team. A high responder for one form of training response (e.g., speed) may not necessarily be a high responder for a different form of training or competition demand (e.g., drive the ball successfully to the basket). Researcher approaches accounting for multivariate, holistic, and flexible factors and variables need to be developed rather than isolated analysis of individual’s performances (e.g., anthropometry). Furthermore, a better understanding of how anthropometrical, maturational, physical, or game-related variables correlate across different age groups would be particularly beneficial given that these factors could be managed to improve the training optimal effects.

Globally, the results of this study confirm a greater importance of the maturational aspects during initial and intermediate stages of boys’ development and performance. Considering the fact that boys in advanced maturity status (both in U-13 and U-15) outscored their counterparts in body height and mass, this may possibly result in a game dominance. Conversely, girls’ clusters showed a lower individual’s variation among them, which apparently suggests somehow a detraction from the importance of maturational aspects in performance evolution. Interestingly, in some cases, best performances were obtained by the players grouped in C3, what could suggest that approximately 1.5 years after the PHV, advantages resulted from maturational bias seem to attenuate. This fact may suggest that the transition between these age groups is less detrimental in performance. Biological maturation can play a large influence on sports selection and talent development (Arede et al., 2019a,b). Within each age category and gender, there is a significant individual variability in terms of growth and maturation, which interacts differently with human performance (e.g., physical and game skills). Thus, if practitioners consider training and competition with respect to the maturational status, it could increase the effectiveness of their decisions, providing more appropriate and individualized stimuli (Cumming et al., 2017; Malina et al., 2019). This approach may include long-term benefits providing the same opportunity and quality of training and competition, irrespectively of current global performance, and suitability to obtain short-term objectives. The present study has some limitations that must be acknowledged. It could be possible that the small size of certain groups may have affected the between-group differences. Moreover, the spectrum of contributing variables is broader; then future studies can include perceptual-cognitive and psychological variables in experimental designs, but also training experience as confound variable. Nevertheless, the findings of the present study do provide new insights in the development of young basketball players. Future research should include a comparison of game performance profiles under different conditions: when playing traditional (i.e., CA-based) and bio-banding (i.e., biological age-based) competitions.

## Conclusion

The results of this study improve our understanding how biological maturation influences both physical and game-related variables in each age category among youth basketball players. Present findings demonstrated the existence of different maturity status within each age category, and greater differences in terms of MO in younger age categories. Moreover, the results of this study have not confirmed a significant difference between maturity status in physical and game-related variables, but they cannot be overlooked. In fact, RGCCA revealed different relative contributions of maturational aspects according to each age category and gender. That said, CA-based training and competition systems may be not enough to provide an individualized stimulus considering growth and maturation. Thus, the strategy of grouping athletes relative to attributes associated with the processes of maturation can be an alternative or complementary approach to provide optimal training/competitive situations, matching individual needs, and consequently encourage long-term development. Practitioners in youth basketball (e.g. coaches, strength and conditioning coaches/athletic trainers, managers, etc.) can use present findings for better matching athletes based on maturity status, according context (e.g., training, competition or strength, and conditioning), and purpose (i.e., talent identification or development).

## Data Availability Statement

The raw data supporting the conclusions of this article will be made available by the authors, without undue reservation.

## Ethics Statement

The studies involving human participants were reviewed and approved by the University of Trás-os-Montes and Alto Douro research ethics committee. Written informed consent to participate in this study was provided by the participants’ legal guardian/next of kin.

## Author Contributions

JA and NL contributed to conceptualization, investigation, methodology, and writing—original draft. JA, IO, and NL contributed to data curation, formal analysis, and software. JA, M-AÁ, and NL contributed to writing—review and editing. All authors contributed to the article and approved the submitted version.

## Conflict of Interest

The authors declare that the research was conducted in the absence of any commercial or financial relationships that could be construed as a potential conflict of interest.
